# Cancer: What It It? Causes and Treatment

**Published:** 1892-02

**Authors:** C. S. Reeves

**Affiliations:** Longrove, Texas


### Correspondence.

### Cancer—What is It? Causes and Treatment.

BY C. S. REEVES, M. D., LONGROVE, TEXAS.

LONE GROVE, LLANO COUNTY, TEXAS, January 12, 1892.

*Editor Daniel's Texas Medical Journal:*

This being the concluding article on cancer, we merely sum up conclusions:

1. Cancer is often (not always) hereditary.
2. It is very like phthisis pulmonalis in its *general* character.
3. The bacteria of both diseases are probably very near akin.
4. Many sores, at first easily cured; by failure of proper treatment, afterwards become cancerous, for the same reasons that a neglected "bad cold" leads to a cough, sore throat, bronchitis, asthma, pneumonia, consumption.
5. If these cancerous deposits invite the bacteria from without, as in the case of the blow-fly, it can *never* be possible for the surgeon's knife to effect a *radical cure*, after these have had sufficient time to penetrate the system through the circulation of the blood.
6. From these considerations, I, like the immortal Gross, *might* call in the aid of the surgeon to amputate a finger or toe, but not under any other consideration.
7. Cancer may be *cured* by the administration of tonics and local application, if taken before the vital powers of the system have given away.
8. From these data I have formulated what I conceive to be a rational treatment, which I purpose bringing before the profession in a *professional way*, at the next annual meeting of the Texas Medical Association. Meantime I would add, that the new application of electricity to the disease under consideration, is destined ere long to revolutionize our treatment in this as well as many other forms of disease.

